# A Case of Morphine Poisoning Apparently Relieved by Atropine

**Published:** 1869-11

**Authors:** J. F. Miner


ART. III.—*A case of Morphine Poisoning apparently relieved by Atropine.* By J. F. MINER, M. D.

A strong, vigorous man, while suffering from temporary alcoholic excitement, swallowed three grains of morphine, receiving it from the hands of a druggist, and in his presence taking it. He now declares his purpose, and says: "I shall be a dead man in three hours." He gets into his carriage and, with his companion, drives off. The alarmed druggist calls immediately upon me and states the facts, desiring my attendance. Upon reaching the gentleman's residence we found that he had just returned, and was sitting in his chair, resolutely refusing all attentions, and saying that the thing was done, with due consideration, for the purpose of getting out of the

*CORRECTIONS.—In Prof. Rochester's Clinic, as published in the October number, on the 82d page, 17th line, for weakness read restlessness. In the formula for Sat. Sol Potass. Chlor. ℥ iii., read ℥ viii. There were, likewise, errors in Cases II. and III.

world; wanted to know if I thought the quantity was sufficient. Said he had taken three grains before leaving home, and wanted to know how much he had taken in the store. About half an hour had now intervened since taking the morphine, which was done at very near three o'clock in the afternoon. An emetic was obtained and urged upon him, but to no avail; it was at this time impossible to do anything for him or with him. At 4½ o'clock he consented to take strong coffee, of which he continued to drink freely for five or six hours, at all times when he could be sufficiently aroused. At 5 o'clock the narcotism was very apparent, but he conversed with considerable consciousness, still inquiring if the dose was "sufficient." He now took one-fourth grain atropine without resistance, and in about half an hour one-tenth grain more. At 6 o'clock it was difficult to keep him awake; would walk with the help of assistants on each side, but slept walking. Pupils were contracted to a point; pulse 120; respiration not very slow or labored.

*Seven o'clock.*—Narcotism almost complete; could only be aroused enough to continue respiration, which would cease if allowed to remain undisturbed. Fluids placed in the mouth remained unswallowed, and were inhaled rather than swallowed, causing cough and great difficulty of breathing. The face was swollen and of a dark livid hue, conjunctiva injected, and all the vessels of the lids distended. The pupils were observed to be less contracted than they had been for the preceeding two hours; no other effects of atropine observed unless the thirst and dryness of the tongue was due to this agent.

*Eight o'clock.*—General appearance and most of the symptoms unchanged. With great difficulty could be reminded of the necessity of continuing respiration, which, if left to himself, would, after a few respirations, cease; violent shaking and smart blows would arouse sufficiently to make him continue breathing. Pupils now of natural size; compared with others in the same light, they were fully as large.

*Nine o'clock.*—More easily aroused and made to answer when violently shaken; when raised upon his feet made some effort to stand. Pupils not greatly changed, but seemed to be a little more dilated.

*Eleven o'clock.*—Had so much improved in appearance and symptoms, that all danger seemed passed, and he was left with competent assistants for the night.

The interest, if any, which this case possesses, is in the combined action of the two agents—morphine and atropine. It is claimed that atropine is an antidote for morphine, and this claim has been very well sustained by some carefully made experiments upon animals. Dr. Percy, of New York, has written an instructive monograph upon the subject, in which he describes some well conducted experiments, showing very conclusively that, at least, when given to animals, these drugs have antidotal properties. Many physiologists have also experimented considerably with the view of determining the value of atropia as an antidote for morphia; I should perhaps say, with varying results. It is not common to have opportunity to test in man the effects of poisonous doses of these two agents. I confess to a fear of giving unsafe quantities of atropine, even though the patient had taken "sufficient" morphine to prove fatal in most instances. If he had taken, as he declares, three grains before leaving home, he certainly had "sufficient" for fatal effects; but, if only three grains in all, still the dose was "sufficiently" unsafe, possibly, however, not fatal. The atropine in this case produced no positive effects which can be certainly traced, unless it be its specific action upon the iris. That it did act to dilate the pupil at a time when otherwise it would have remained contracted, I have no doubt, but I am not *certain* that it had any effect to render the narcotism less profound. It seems probable that men accustomed to alcoholic stimulants can bear larger doses of the narcotics without fatal effects than men of temperate habits, but of this there may be differences of opinion.

This experiment was not a well conducted one, as all will observe, for determining this important question; it was not instituted for this purpose. I was willing to appropriate as much of it as could bear upon the question, but was not prepared to extend it for the purpose of observation. I have related the facts as suggestive at least of the value of atropine in the treatment of narcotic poisoning, but many similar opportunities are necessary to positively determine any of the points at issue.

